# Application of Trap Cropping as Companion Plants for the Management of Agricultural Pests: A Review

**DOI:** 10.3390/insects9040128

**Published:** 2018-09-25

**Authors:** Shovon Chandra Sarkar, Endong Wang, Shengyong Wu, Zhongren Lei

**Affiliations:** State Key Laboratory for Biology of Plant Diseases and Insect Pests, Institute of Plant Protection, Chinese Academy of Agricultural Sciences, Beijing 100193, China; shovon47@gmail.com (S.C.S.); edwang@ippcaas.cn (E.W.)

**Keywords:** cultural control, biological-based control, integrated pest management, natural enemy

## Abstract

Companion planting is a well-known strategy to manage insect pests and support a natural enemy population through vegetative diversification. Trap cropping is one such type of special companion planting strategy that is traditionally used for insect pest management through vegetative diversification used to attract insect pests away from the main crops during a critical time period by providing them an alternative preferred choice. Trap crops not only attract the insects for feeding and oviposition, but also act as a sink for any pathogen that may be a vector. Considerable research has been conducted on different trap crops as companion plant species to develop improved pest management strategies. Despite this, little consensus exists regarding optimal trap cropping systems for diverse pest management situations. An advantage of trap cropping over an artificially released natural enemy-based biological control could be an attractive remedy for natural enemies in cropping systems. Besides, many trap crop species can conserve natural enemies. This secondary effect of attracting natural enemies may be an advantage compared to the conventional means of pest control. However, this additional consideration requires a more knowledge-intensive background to designing an effective trap cropping system. We have provided information based on different trap crops as companion plant, their functions and an updated list of trap cropping applications to attract insect pests and natural enemies that should be proven as helpful in future trap cropping endeavors.

## 1. Introduction

Conventional agricultural practices have detrimental effects on the environment, human health and food security, including pesticide contamination of food, insect pest resistance to insecticides and the harm of non-target organisms, including pollinators and beneficial insects [1], resulting in a shift to alternative management strategies, namely biological control for insect pests [2]. Conservation biological control through vegetative diversification is an effective strategy for pest management. Several conservation biological control practices, such as “farmscaping”, have gained popularity in pest control due to their ability to fulfill essential criteria like efficacy, predictability and cost [3,4,5]. Trap cropping is a means of promising conservation biological control that involves growing another non-crop species in a selected area to attract pests from target crop, preventing pests from reaching the crop and finally to control that pest in order to reduce damage to the main crop [6,7]. Since the 1930s, there have been numerous reported cases of successful trap cropping for managing various insect pests, ultimately resulting in a substantial reduction in the use of pesticides in developing countries [6]. However, with high densities of pests on these new trap plants placed within agricultural fields, preventing insect pest dispersal from the trap plants back on to the focal crop is essential for trap cropping to provide meaningful pest control [8]. In fact, every successful trap cropping example at a commercial scale has included some method to either reduce this dispersal by either increasing trap crop retention of pests or increasing pest mortality on the trap crop [8]. Biological control is one especially promising way to increase pest mortality on the trap crop, without having to spray the trap crop with pesticides. Fortunately, trap crops can potentially attract natural enemies of insect pests [9,10,11], and through predation and parasitism, these natural enemies reduce the ability of trap crops to act as pest breeding grounds to disperse back into the main crop.

Therefore, understanding the interactions between trap cropping and natural enemies may be essential to the future success of trap cropping systems. In this paper, we have reviewed the trap cropping literature with a focus on its potential to enhance biological control.

## 2. Function of the Trap Cropping System in Agriculture

### 2.1. Description of the Trap Cropping System

In organic crop production, pest management relies primarily on habitat manipulation through farmscaping and other biological control practices [12]. It has been observed that polycultures of crop species often lead to less damage from pests than monocultures of crops within a given area [13,14]. One explanation for this was proposed by Root [15], that polycultures can enhance biological control by offering greater host capacity for natural enemies while simultaneously complicating the pest habitat. A habitat manipulation through trap cropping capitalizes on the strong perimeter-driven behavior in multiple cropping systems [16,17,18,19].

Trap cropping is an attractive option to reduce dependency on conventional pest management practices through insecticides. Indeed, insecticides are costly and hazardous (even the organic ones), and some insect pests have developed resistance against them [20]. An example could be the stink bug (Hemiptera: Pentatomidae), which can further be exacerbated by their long life cycle, high capacity to disperse and polyphagous nature leading to a landscape-wide agro-ecosystem threat. Trap crops have been shown to effectively manage stink bugs (*Halyomorpha halys* Stål (Hemiptera: Pentatomidae)) in conventional and organic crop production systems [8,19]. Sorghum (*Sorghum bicolor* L.) has been successfully used as trap crop in cotton fields [17]. Similarly, black mustard reduced kernel injury by 22% in sweet corn caused by *Nezara viridula* L. (Heteroptera: Pentatomidae) [21]. An efficient trap crop system should have at least double the pest attraction capacity of the cash crop during its vulnerable stage with an easy management strategy and should cover no more than 2%–10% of the total crop area [7,8].

### 2.2. Factors that Affect the Efficacy and Practicality of Trap Cropping Systems

The basic factors for a successful trap cropping system are trap crop species and their spatial arrangement. In the following sections, we have discussed existing research that may benefit the development of trap cropping systems.

Different trap crop species can release different types of volatile compounds due to a specific elicitor that can attract insects [22]. Similarly, trap crop volatiles can attract and enhance the foraging efficacy of natural enemies in an agro-ecosystem. For example, volatiles emitted by several plants can attract green lacewing (Neuroptera: Chrysopidae) [23,24]. The behavioral response of pollen beetle (*Meligethes aeneus* Fabricius (Coleoptera: Nitidulidae)) is enhanced by volatiles released from turnip rape (*Brassica rapa* L. (Brassicaceae)) and oilseed rape (*Brassica napus* L. (Brassicaceae)) [25]. A multi-compound blend is more attractive than a single chemical constituent because herbivorous insect pests often locate and choose hosts using a blend of chemical cues [26,27]. Therefore, pairing of trap crop species might provide a long-term effect of attracting insect pests such that the host plants often vary in their chemical profiles through time [28,29]. For example, pestiferous beetles and bugs can be intercepted and arrested by highly attractive squash varieties for controlling herbivores and thus largely restrict pest damage for cucumber, butternut squash or watermelon crop production [1,30,31,32]. Successful reduction of noctuid moth’s (Lepidoptera: Noctuidae) infestation is possible when corn attracts them from vegetable crops and retains them as a trap crop [33,34].

Trap crop species are highly attractive to pests species and are inter-planted with susceptible crops, which can attract and divert pests from the main crop. This practice relies on the exploitation of insect preferences for certain host plants, based on visual, tactile or olfactory cues [35,36]. Therefore, in both long- and short-range host identification, pairing of a chemically attractive trap crop species with a second trap species that provides visual or tactile cues might more effectively draw in pests than either species alone [37]. Many different plant species have been tested to develop trap cropping systems. It is necessary that trap crop species have the same horticultural requirements of the crop with which they are grown, including similar light and temperature demands. For example, mung bean (*Vigna radiata* (L.) R. Wilczek (Fabaceae)) has been shown to be an effective trap crop for *Apolygus lucorum* Meyer-Dür (Hemiptera: Miridae) and is gradually being adopted for control of this pest in cotton fields in northern China [38]. Resistant cultivars of trap crops to improve health and longevity that can grow rapidly and inexpensively are also important factors. Sunflowers *Helianthus* spp. (Asteraceae) are a particularly pest-resistant trap crop option, as they have been used with success as attractive plants for coleopteran, lepidopteran and hemipteran pests [7,8].

The spatio-temporal arrangement of trap crop around the main crop is one of the vital factors for its effectiveness. There are many strategies for arranging a trap crop system. For example, Smyth et al. [39] recommended planting trap crops sequentially with main crops so that the attractive phenological stage of both crops can be presented at the same time. On the other hand, Potting et al. [34] reported that a trap crop with a border arrangement is the best arrangement. Field margin manipulation using a more attractive plant is quite common in integrated pest management programs. The strong perimeter-driven behavior of the brown marmorated stink bug (*H. halys*) and the brown stink bug (*Euschistus servus* Say (Hemiptera: Pentatomidae)) could potentially be increased by raising a highly attractive trap crop border in a perimeter surrounding the cash crop [7,15,16,17]. However, this practice does not always provide the best results [40]. In a previous study, *Heliothis zea* Boddie (Lepidoptera: Noctuidae) infestation was substantially reduced when an upwind corn border with fresh silks was used as a trap crop compared to fields with no corn border in a tomato (*Solanum lycopersicum* L. (Solanaceae)) field [41]. Sequential trap crops are cultivated prior to or after the main crop [7]. For example, trap crop planted before planting sugar beets on more than 40% of the German sugar beet cropping areas primarily to reduce the nematode population leads to improve yield [42]. In Finland, multiple trap crop species (Chinese cabbage (*Brassica oleracea*), marigolds (Asteraceae), rapes (Brassicaceae) and sunflower (Asteraceae)) have been used in cauliflower (*Brassica oleracea* L. (Brassicaceae)) fields for successful control of rape blossom beetle (*Meligethes aeneus* Fabricius (Coleoptera: Nitidulidae)) [43]. Trap crop (perimeter) also can be combined with a repellent intercrop to develop a push-pull strategy for insect pests management. In Kenya, Khan et al. [44] reported that maize stem borer (*Busseola fusca* Fuller (Lepidoptera: Noctuidae)) can effectively be controlled by using a push-pull strategy, when *Desmodium* grasses (Fabaceae) are intercropped with Napier grass (*Pennisetum purpureum* Schumach (Poaceae)) planted as a perimeter trap crop.

## 3. Trap Cropping in Insect Pest Attraction and Repulsion

### 3.1. Trap Cropping in Insect Pest Management

**Figure 1:**
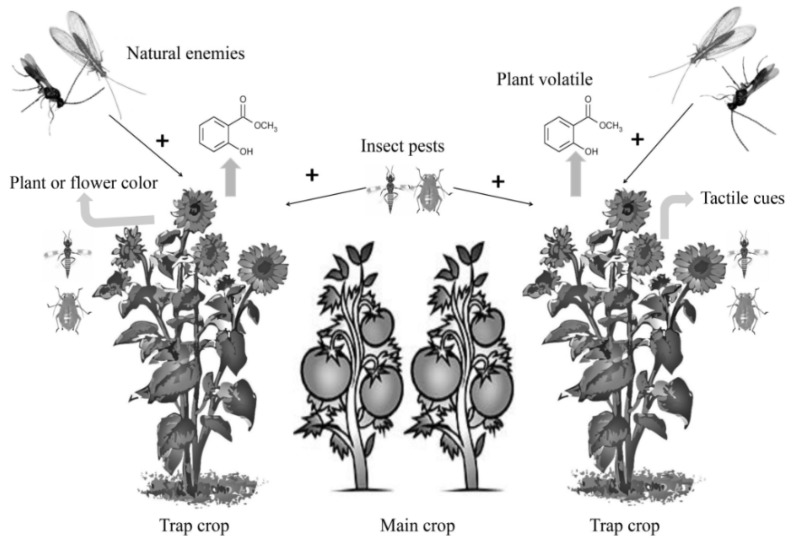
Role of trap crop to attract insect pests and natural enemies in a farming system.

A trap crop system is usually designed to attract agricultural pests, usually insects, away from the main crop (Figure 1). For example, in an onion (*Allium cepa* L. (Amaryllidaceae)) field, populations of *Thrips tabaci* Lindeman (Thysanoptera: Thripidae), a major pest of onion, can be suppressed by trap crop buckwheat (*Fagopyrum esculentum* Moench (Polygonaceae)) [45]. Furthermore, a combination of two trap crop species can attract insect pests more effectively, such as sunflower (Asteraceae) and grain sorghum (Poaceae) planted to attract the brown marmorated stink bug (*H. halys*) from bell peppers [16,46] (Table 1). Indeed, the successive planting of second trap crops can extend the period of attractiveness for insect pests [47].

Several cruciferous crops have been tested as trap crops. Srinivasan and Moorthy [48] tested Indian mustard (*Brassica juncea* (L.) Czern. (Brassicaceae)) as a trap crop to aid in managing the major lepidopterous pests *Plutella xylostella* L. (Lepidoptera: Plutellidae) and *Crocidolomia binotalis* Zeller (Lepidoptera: Crambidae) on cabbage, and Charleston and Kfir [49] also reported that, as a trap crop, Indian mustard (*B. juncea*) can attract diamondback moths (*Plutella xylostella* L. (Lepidoptera: Plutellidae)) from several economically important cruciferous crops. A similar outcome was found in recent trap crop research, when Indian mustard (*B. juncea*) was protected by Ethiopian mustard (*Brassica carinata* A. Braun (Brassicales: Brassicaceae)) as a trap crop to control *Pieris brassicae* L. (Lepidoptera: Pieridae) [50]. Furthermore, to suppress flea beetle *Phyllotreta* spp. (Coleoptera: Chrysomelidae) infestation, Chinese cabbage was planted with white cabbage as trap crop [51]. All studies resulted in pest populations being reduced when trap crops were present compared to those without trap crops.

Only a few successful cases of trap crop application have been conducted at the commercial level targeting mainly Coleoptera, Hemiptera and Lepidoptera species. These cases involved insects that directed their movement and tended to aggregate on a highly attractive trap crop [7]. In addition, trap cropping is mostly effective against flying insects. Development of trap crop systems that require only plants to provide pollen or another resource may simplify implementation and maintenance. For example, eggs and larvae of the imported cabbage worm (*Pieris rapae* L. (Lepidoptera: Pieridae)), larvae of the diamondback moth (*P. xylostella*) and larvae of the cabbage looper (*Trichoplusia ni* Hübner (Lepidoptera: Noctuidae)) were more abundant in nectar-producing plants inter-planted with broccoli (*Brassica oleracea* L. (Brassicaceae)) [52]. According to Banks and Ekbom [53], for a successful trap cropping system, a very important factor is the attractiveness of the trap crop and its proportion in the field. Even the use of a highly attractive trap crop may not be successful if their percentage is too low to be effective. The proportion of these two factors is critical in deploying a successful and effective trap cropping system. However, crop species have not been systematically evaluated for their effect on the growth of alternative hosts and natural enemies. Crop species of low quality for herbivores due to low nutritional values or high defenses can reduce herbivore development and reproduction [54].

Usually, trap crop efficiency greatly depends on the additional pest management practices, and not all insects can be controlled with trap cropping. Application of trap cropping is not a foolproof solution to all pest problems because it does require additional pest management skills and a thorough understanding of insect behavior. The effectiveness of trap crops can be increased by supplemental use of other control methods, such as targeted insecticide sprays and vacuuming [7,8]. Castle [55] suggested to apply insecticides to control *Bemisia tabaci* Gennadius (Hemiptera: Aleyrodidae) on a cantaloupe (*Cucumis melo* var. cantalupo Ser. (Cucurbitaceae)) trap crop, thereby preventing adult dispersal into the main cotton crop. However, researchers exploring wider ecological functions, such as simultaneously controlling multiple pests, protecting natural enemies and enhancing their biological effectiveness, may help to accelerate the use of trap cropping in insect pest management.

### 3.2. Trap Cropping in Natural Enemy Attraction

Conservation of natural enemies is one of the attractive biological control tactics used in most agro-ecosystems; though, in most of the cropping systems, natural enemies are usually one step behind the pests [74,75,76]. In the case of annual crops, the most useful mechanism for conservation biological control is spatial attraction of natural enemies resulting in a near linear decline in pest density [77], and trap crop could be an attractive option to attract them.

A plant species able to attract simultaneously both pests and their natural enemies can be used in a trap cropping system (Figure 1) for conservation biological control program. For example, trap plant Borage *Borago officinalis* L. (Boraginaceae) has been found to be attractive to the herbivorous Aphididae and two aphid-controlling bio-agents, parasitoid *Aphidius colemani* Viereck (Hymenoptera: Braconidae) and Chrysopidae predator species [78,79]. Indeed the complementary effect suggests that multiple natural enemies would strengthen pest control (e.g., Williams et al. [80] found that the combination of two generalist natural enemies, such as green lacewings (Neuroptera: Chrysopidae) and lady beetles (Coleoptera: Coccinellidae), which are attracted to a plant volatile (methyl jasmonate) in cotton fields, was found to lead to the complementary control of insect pests). Moreover, a potential secondary trap plant might help to improve the efficacy of natural enemies. For example, the average infestation rate of pods by *Ceutorhynchus obstrictus* Marsham (Coleoptera: Curculionidae) was below 10% for plants in the cruciferous family: *B. rapa*; *Sinapis alba* L. (Brassicaceae) and *B. juncea* when planted with *B. napus* [81]. Furthermore, integration of additional technological tools can play an important role in pest suppression by attracting natural enemy species.

### 3.3. Technological Tool for Trap Cropping to Improve Natural Enemy Attraction

Attraction of natural enemies by behavioral manipulation is not a new topic [82,83,84,85,86]. To monitor major pests in agriculture and forest environment, commonly, sex pheromone has been used as an attractant for a long period of time. Another option is to make the plant more attractive to pests and natural enemies. Different volatiles can play important roles in attracting natural enemy species. Male and female *Chrysoperla carnea* Stephens (Neuroptera: Chrysopidae) adults will respond to several semiochemicals produced by corn (*Zea mays* L. (Poaceae)), as well as a prey species of aphid (*Acyrthosiphon pisum* Harris (Homoptera: Aphididae)) [24]. Rhino et al. [41] used corn as a potential trap crop for *Helicoverpa zea* Boddie (Lepidoptera: Noctuidae). Therefore, semiochemicals emitted from trap plant and pest species could be attractive for natural enemies. Moreover, herbivore-induced trap crops can emit herbivore-induced plant volatiles (HIPVs) and use a catalyst to control agricultural pests by attracting natural enemies [87,88,89,90,91,92,93,94]. For example, an aphid infested borage plant can attract *Aphidius colemani* Viereck (Hymenoptera: Braconidae) [60]. A plant volatile or a mixture of plant volatiles can also improve natural enemy attraction capacity in a trap cropping system. For instance, methyl salicylate is used as a common attractant for natural enemies and insect pests. Both synthesized and methyl salicylate released from herbivore-induced plant volatile has been shown to be attractive to green lacewings [64,95,96]. Alfalfa could be an example, which can release attractive plant volatiles for natural enemies. Zhu et al. [97] observed a high abundance of green lacewing adults in early summer in alfalfa (*Medicago sativa* L. (Fabales: Fabaceae)) fields (Table 2). However, the value of using plant volatiles to manipulate natural enemies is still unclear [98].

A sugar-rich food source is needed for the egg parasitoid *Telenomus laeviceps* Förster (Hymenoptera: Scelionidae) to increase their parasitization performance and female offspring abundance [99]. In that case, artificial food-spray could be an additional resource for beneficial insects. Thereafter, the use of an artificial food spray remains a possibility in conservation biological control [100], because it can attract and intercept natural enemies in an area [101,102]. More than 50 years ago, the first documented field usages of artificial food sprays occurred with sucrose solutions [103,104]. Many natural enemies are attracted to plant-derived foods such as pollen, nectar, extra-floral nectar or honeydew in their immature and adult diets [105,106,107]. However, artificial food spray in trap crop to attract natural enemies is necessary to improve through a better understanding of the ecological basis.

While trap crop is an attractive option for organic crop production through attraction of natural enemies, it also has many limitations. Depending on target insect pests and the cropping system, only a few programs involve trap crops to attract natural enemies [7]. Besides, some programs introduced new species of natural enemies in trap crops, which often disrupts the ecosystem by competing with native natural enemy species, and only a few of them are known to switch their target insect host [114].

Trap cropping to attract natural enemies is knowledge intensive to maximize its effectiveness. Many crops are infested by multiple arthropod pest species, often making it impossible to control with one natural enemy within a given ecosystem. Moreover, the process of actually developing a successful trap crop system is costly and, in most cases, involves a secondary product with little, if any, market value [10]. Although biocontrol with natural enemies may ultimately have a desirable long-term effect, achieving the desired results is usually a slow process requiring considerably more time than simply applying pesticides on trap crops.

## 4. Conclusions

It is true that there has been a long history of research involving trap crops, with researchers investigating many pest species (Table 1) and natural enemy species that are attracted to prospective trap crops (Table 2); still, little consensus exists regarding an optimal trap cropping system. There is no specific recommendation of attractive plant to a specific pest, as well as their natural enemy.

According to Gurr et al. [115], attainment through biological control can be measured as, “whether crop damage is reduced to the extent that adequate control—usually regarded as maintenance below the economic injury level—is afforded, and whether significant proportions of farmers adopt this approach to pest management”. For establishing a successful trap cropping system in different agronomic situations, primarily a thorough understanding is required of the behavior and preferences of the targeted pests, as well as the dispersal and the attraction of natural enemies for the trap crop species. There still exists a need for basic biological and ecological research on the specific host plants involved, along with their pests, appropriate trap crops and their natural enemies, especially their interactions with each other, to improve and implement successful biological control programs using trap crop systems. Further study is also needed on the effective application of trap crops, including cropping pattern (e.g., perimeter, sequential, multiple and push-pull planting schemes), the total percentages compared to the cash crop, as well as maintenance details.

This review has highlighted several potential advantages of using trap cropping systems that may make them more important and economical. The success and implementation of biological control with trap crop will be increased, if future research demonstrates the long-term, preventive and economically efficient way to control insect pests. However, in the present situation, attraction of natural enemies by the trap crop and continuous production of them may be counter-acted by more cost effectiveness. In our opinion, trap crop species that have natural enemy attracting capacity will be greatly enhanced if future research works are conducted with diverse concepts and modalities.

## Figures and Tables

**Table 1:** Research and demonstration projects that have implemented trap cropping systems to attract insect pests in ornamental and food crops.

Trap Crop	Crop	Insect Pest	Country	Implementation	References
African marigold, *Tagetes erecta* L. (Asteraceae)	Tomato, *Solanum lycopersicum* L. (Solanaceae)	*Helicoverpa armigera* Hübner (Lepidoptera: Noctuidae)	India	Field	Srinivasan et al. [56]
Alfalfa, *Medicago sativa* L. (Fabaceae)	Lettuce, *Lactuca sativa* L. (Asteraceae)	*Lygus rugulipennis* Hahn (Hemiptera: Miridae)	Italy	Field	Accinelli et al. [57]
Arugula, *Eruca sativa* Mill. (Brassicaceae);	Tomato, *S. lycopersicum*	*Lygus* spp. (Hemiptera: Miridae)	United States	Field	Swezey et al. [58]
Buckwheat, *Fagopyrum esculentum* Moench (Polygonaceae)	Onion, *Allium cepa* L. (Amaryllidaceae)	*Thrips tabaci* Lindeman (Thysanoptera: Thripidae)	United States	Field	Buckland et al. [45]
Buttercup squash, *Cucurbita maxima* Duchesne (Cucurbitaceae)	Athena muskmelon, *Cucumis melo* L. (Cucurbitaceae)	*Acalymma vittatum* (Coleoptera: Chrysomelidae); *Diabrotica undecimpunctata* L. (Coleoptera: Chrysomelidae)	United States	Field	Cavanagh et al. [59]
	Cucumber, *Cucumis sativus* L. (Cucurbitaceae)	*Acalymma vittatum* (Coleoptera: Chrysomelidae)	United States	Field	Adler and Hazzard [1]
Carrot, *Daucus carota* Hoffm. (Apiaceae)	Onion, *A. cepa*	*Thrips tabaci Lindeman* (Thysanoptera: Thripidae)	United States	Field	Buckland et al. [45]
Chinese cabbage, *Brassica rapa* L. (Brassicaceae)	White cabbage, *Brassica oleracea* L. (Brassicaceae)	*Phyllotreta spp.* (Coleoptera: Chrysomelidae)	Slovenia	Field	Trdan et al. [51]
Collard cabbage, *Brassica oleracea* viridis (Brassicaceae)	Cabbage, *B. oleracea*	*Plutella xylostella* L. (Lepidoptera: Plutellidae)	United States	Field	Mitchell et al. [60] Shelton and Nault [61]
Eggplant, *Solanum melongena* L. (Solanaceae);	Common bean, *Phaseolus vulgaris* L. (Fabaceae)	*Bemisia argentifolii* Gennadius (Hemiptera: Aleyrodidae)	United States	Field	Smith and Mcsorley [62]
Ethiopian mustard, *Brassica carinata* A.Braun (Brassicaceae)	Indian mustard, *Brassica juncea* (L.) Czern. (Brassicaceae)	*Pieris brassicae* L. (Lepidoptera: Pieridae)	India	Laboratory and field	Kumar [50]
Indian mustard, *B. juncea*	Cabbage, *B. oleracea*	*Plutella xylostella* L. (Lepidoptera: Plutellidae); *Crocidolomia binotalis* Fabricius (Lepidoptera: Crambidae)	India	Laboratory and field	Srinivasan and Moorthy [48]
	Crucifer crops, *Brassicaceae* spp. (Brassicaceae)	*Plutella xylostella* L. (Lepidoptera: Plutellidae)	South Africa	Field	Charleston and Kfir [49]
Marigold, *Calendula officinalis* L. (Asteraceae)	Tomato, *S. lycopersicum*	*Helicoverpa armigera* Hübner (Lepidoptera: Noctuidae)	India	Laboratory and field	Kumar et al. [63]
Mung bean, *Vigna radiata* L. (Fabaceae)	*Bacillus thuringiensis* (Bt) cotton, (Bt) *Gossypium hirsutum* L. (Malvaceae)	*Apolygus lucorum* Meyer-Dür (Hemiptera: Heteroptera )	China	Field	Lu et al. [38]
Napier grass, *Pennisetum purpureum* Schumach (Poaceae)	Sorghum, *Sorghum bicolor* L. (Poaceae)	*Busseola fusca* Fuller (Lepidoptera: Noctuidae)	United States	Field	Khan et al. [64]
Non-flowering *Barbarea*, *Barbarea* spp. (Brassicaceae)	Cabbage, *B. oleracea*	*Plutella xylostella* L. (Lepidoptera: Plutellidae)	Spain	Field	Badenes-Pérez et al. [65]
Sorghum, *S. bicolor*	Maize, *Zea mays* L. (Poaceae)	*Chilo partellus* Swinhoe (Lepidoptera: Crambidae)	Kenya	Field	Midega et al. [66]
	Cotton, *G. hirsutum*	*Nezara viridula* L. (Hemiptera: Pentatomidae)	United States	Field	Tillman [67]
Summer squash, *Cucurbita pepo* L. (Cucurbitaceae)	Bean, *P. vulgaris*	*Bemisia argentifolii* Gennadius (Hemiptera: Aleyrodidae)	United States	Field	Smith et al. [68]
Sunflower, *Helianthus annuus* L. (Asteraceae); grain sorghum, *S. bicolor*	Bell peppers, *Capsicum annuum* L. (Solanaceae)	*Halyomorpha halys* Stål (Hemiptera: Pentatomidae)	United States	Field	Blaauw et al. [16,46]
Yellow rocket, *Barbarea vulgaris* W. T. Aiton (Brassicaceae)	Cabbage, *B. oleracea*	*Plutella xylostella* L. (Lepidoptera: Plutellidae)	United States	Field	Badenes-Perez et al. [69]
Indian mustard, *B. juncea*; white mustard, *Sinapis alba* L. (Brassicaceae)	Chinese Cabbage, *B. rapa*; Oilseed rape, *Brassica napus* L. (Brassicaceae)	*Ceutorhynchus obstrictus* Marsham (Coleoptera: Curculionidae)	Estonia	Field	Kovács et al. [70]
Black mustard, *Brassica nigra* L. (Brassicaceae); radish, *Raphanus sativus Pers.* (Brassicaceae); arugula, *E. sativa*	Oilseed rape, *B. napus*	*Meligethes aeneus* Fabricius (Coleoptera: Nitidulidae)	Estonia	Field	Kaasik et al. [71]
Oilseed rape, *B. napus*; Chinese cabbage, *B. rapa*; black mustard, *B. nigra*; Indian mustard, *B. juncea*	White mustard, *S. alba*; Radish, *R. sativus*	*M. aeneus*	Estonia	Field	Veromann et al. [72,73]

**Table 2:** Research and demonstration projects that have implemented cropping systems to attract natural enemies in ornamental and food crops.

Trap Crop	Crop	Natural Enemy	Country	Implementation	Reference
Alfalfa, *M. sativa* L.	Maize, *Z. mays*	*Chrysopidae*	United States	Field	Zhu et al. [97]
Borage, *Borago officinalis* L. (Boraginaceae)	Tomatoes, *S. lycopersicum*	*A. colemani*; *Syrphidae*; *Chrysopidae*	Japan, United States	Greenhouse, field	Fujinuma et al. [78] Hogg et al. [108]
Coriander, *Coriandrum sativum* L. (Apiaceae);	Banana, *Musa balbisiana* L. (Musaceae)	*Chrysopidae*; *Coleomegilla maculata* De Geer (Coleoptera: Coccinellidae)	Brazil	Greenhouse	Salamanca et al. [109]
Cornflower, *Centaurea cyanus* L. (Asteraceae)	Squash, *C. pepo*	Spiders; *Carabidae*	United States	Field	Fair and Braman [110]
Maize, *Z. mays*	Cucumber, *C. sativus*	*A. colemani*	United States	Field	Bennison and Corless [111]
Sunflower, *H. annuus*	Banana, *M. balbisiana*	*Chrysopidae*	United States	Field	Zhu et al. [97]
Sunflower, *H. annuus*	Cotton, *G. hirsutum*	*Chrysopidae*; *Coccinellidae*	United States	Field	Williams et al. [80]
Sunn hemp, *Crotalaria juncea* L. (Fabaceae)	Tobacco, *Nicotiana tabacum* L. (Solanaceae)	*Crocothemis servilia* Drury (Odonata: Libellulidae); *Orthetrum Sabina* Drury (Odonata: Libellulidae)	Indonesia	Field	Trisnawati and Azis [112]
Sweet alyssum, *Lobularia maritime* L. (Brassicaceae)	Cruciferous vegetables, *Brassica* spp. (Brassicaceae)	*Syrphidae*	United States	Field	Hogg et al. [79]
Wheat, *Triticum aestivum* L. (Poaceae)	Cucumber, *C. sativus*	*A. colemani*	United Kingdom	Greenhouse	Jacobson and Croft [113]
